# Examining the Longitudinal Relationship Between Metacognitive Beliefs and Psychological Distress in an Adolescent Population: A Preliminary Analysis

**DOI:** 10.1007/s10578-023-01611-z

**Published:** 2023-10-13

**Authors:** Katie Schultz, Lee Kannis-Dymand, Daniel Jamieson, Larisa T. McLoughlin, Siobhan Loughnan, Andrew Allen, Daniel F. Hermens

**Affiliations:** 1 School of Health, University of the Sunshine Coast, Maroochydore DC, QLD Australia; 2 Thompson Institute, University of the Sunshine Coast, Birtinya, QLD Australia; 3 Stillbirth Centre of Research Excellence, Mater Research Institute, South Brisbane, QLD Australia

**Keywords:** Metacognition, Metacognitive beliefs, Adolescence, Psychological distress, Longitudinal, Psychopathology

## Abstract

Adolescence is a period marked by significant vulnerability to the onset of mental health concerns. Within adults, the metacognitive model of psychological disorders advocates for the involvement of metacognitive beliefs in the onset, and maintenance, of psychopathology. The current study aimed to assess the applicability of the metacognitive model in adolescence by exploring the relationship, as well as the trajectory, between metacognitive beliefs and psychological distress. The longitudinal prospective cohort study investigated data from a community-based sample of participants aged 12 to 13. Self-report assessment measures of metacognitive beliefs, psychological distress, and somatic distress are reported across four time-points. Baseline assessments are reported for 70 participants, which reduced to 53 participants at time-point four. Correlational analyses demonstrated a significant relationship between overall metacognition, as well as negative metacognitive beliefs, and psychological distress at each of the four time-points. Generalised Estimating Equations found a significant association between metacognitive predictors and psychological distress over the four time-points. These results indicate that negative metacognitive beliefs, positive metacognitive beliefs, metacognitive beliefs related to superstition, punishment, and responsibility, low perceived levels of cognitive confidence and cognitive self-consciousness predict psychological distress over 12 months in adolescents aged 12 to 13. The strongest longitudinal correlational structure was found for the model of negative metacognitive beliefs and psychological distress. These findings provide preliminary evidence for the positive linear relationship between metacognitive beliefs and psychological distress in adolescence. The study provides an important contribution to understanding the role of metacognitive beliefs in the aetiology and perpetuation of psychological distress in adolescence.

## Introduction

Adolescence is a period of significant susceptibility to the onset of mental health problems. Research confers that 50% of adult mental health concerns emerge before the age of 14 [1]. Within Australia, a national study highlighted that one-fifth (19.9%) of adolescents experienced high or very high levels of psychological distress on a screening tool for anxiety and depressive symptoms; with higher rates of psychological distress associated with the occurrence of a psychological disorder [2]. Hickie et al. [3] advocate for identifying pathways to illness models within mental health,this includes capturing attenuated syndromes which can be an early manifestation of psychopathology. Long term adverse side-effects have been found for untreated psychological disorders with onset in adolescence, highlighting the importance of access to early intervention [4]. The current study responds to appeals for early detection and intervention of mental health concerns in youth by exploring the role of metacognitive beliefs.

The metacognitive model of emotional disorder—the self-regulatory executive function model (S-REF; Wells, 2000; [5, 6] upholds that emotional dysregulation and related negative thoughts are perpetuated by four interrelated constructs: the cognitive attentional syndrome (CAS), metacognitive beliefs, executive control, and mental modes [7]. An in-depth account of this model is beyond the scope of this paper (see [5–7],however, the S-REF model coveys that emotional distress results from the CAS, which is regulated by metacognitive beliefs. The CAS consists of repetitive and perseverative thinking (i.e., worry, rumination, threat focused attention, and the use of maladaptive coping behaviours (e.g. avoidance that fail, contributing to a paradoxical effect on continued negative emotional experience [5, 7]. Metacognitive beliefs control and maintain the CAS and are grouped under two key domains: positive metacognitive beliefs and negative metacognitive beliefs [5]. Positive metacognitive beliefs generally occur before the emergence of negative metacognitive beliefs. Positive metacognitive beliefs are related to the advantages or perceived benefits of engaging in worry or rumination,for example, “worrying helps me to stop bad things from happening” [5]. Positive metacognitive beliefs tend to entail worry being viewed as a helpful strategy. This leads to an increased use of worry as a coping strategy. Whereas, negative metacognitive beliefs are related to either the uncontrollability or harmfulness of one’s thoughts [5, 7]. For example, “my worry is uncontrollable”. Consequently, metacognitive beliefs lead to the utlisation of maladaptive metacognitive strategies such as thought suppression and avoidance that maintain worry or rumination and the related anxiety or negative emotion [5–7].

Research suggests that metacognitive beliefs are present in early adolescence [8]. In their validation of the Metacognitive Questionnaire for Adolescents (MCQ-A), Cartwright-Hatton et al. [8] found support for a five-factor model of metacognitive beliefs in adolescents. This included: positive metacognitive beliefs regarding the usefulness of worry (PBW); negative metacognitive beliefs regarding the uncontrollability and dangerousness of worry (NBW); metacognitive beliefs related to low perceived cognitive confidence (CC); metacognitive beliefs linking thoughts to negative outcomes including superstition, punishment, and responsibility (SPR); and metacognitive beliefs related to cognitive self-consciousness (CSC) and the awareness of one’s thoughts. Indeed, metacognitive beliefs are associated with the onset and maintenance of various psychological disorders within adulthood [9–13]. Furthermore, within non-clinical adult populations, a significant association occurs between metacognitive beliefs, perceived stress, anxiety, and depressive symptoms [14].

Research exploring the relationship between metacognitive beliefs and psychological distress, specifically anxiety and depressive disorder symptoms, in the adolescent population, has yielded mixed findings [8, 15–24]. However, it is important to note that these studies varied considerably methodologically, including sample age range (e.g., child versus adolescent groups), type of psychological condition (e.g., obsessive compulsive disorder, various anxiety disorders), the employed metacognitive belief measure (e.g., MCQ-A, Metacognitive Questionnaire for Children), and most were cross-sectional in design. Arguably, the strongest research support highlights the relationship between NBW and psychological symptoms, with research demonstrating associations with anxiety presentations [17, 20, 21], depressive symptoms [16, 17], and obsessive-compulsive symptoms [23, 24].

Comparatively, limited research supports a positive relationship between CSC and CC metacognitive beliefs, and anxiety or depressive disorder symptoms, in adolescents. In fact, research exploring CSC in adolescents indicates the use of these metacognitive beliefs may be a normative process in youth with no differences found between clinical and non-clinical populations [8, 17] or higher endorsement found in non-clinical populations [15, 16]. However, due to the limited studies exploring CSC in youth, further research is required to determine the relationship between monitoring one’s thoughts and psychological distress in adolescents.

Overall, the evidence exploring the applicability of the S-REF model in adolescents is promising. Nevertheless, limited studies, and mixed findings, demonstrate the need for further research. Additionally, many studies consist of mixed child and adolescent populations, likely confounding the research with age-based effects due to the development of cognitive awareness that occurs through childhood and into adolescence [16]. Further, the majority of the studies have consisted of cross-sectional research designs signifying the need for longitudinal analysis [8, 15–17, 19, 20, 22–24].

Notably, there appears to be only two peer-reviewed publications with a longitudinal design that have included adolescent populations. Zhou et al. [25] evaluated 14–18 year old, Chinese high school students (*N* = 313), using the Metacognitive Questionnaire 30 item version for adults (MCQ-30). Using a 6-month longitudinal design, Zhou et al. identified that maladaptive metacognitive beliefs (i.e., total score on the MCQ-30 rather than subscale scores) were predictive of elevated depressive symptoms. Köcher et al. [26] researched 8–16 year olds (*N* = 27) with an anxiety disorder, pre- and post-exposure therapy (11 sessions), using the positive and negative subscales of the German version of the Metacognitive Questionnaire for Children. No significant changes in metacognitive beliefs were found post-treatment; however, negative metacognitive beliefs were significantly correlated with changes in anxiety symptoms. The above studies reveal metacognitive beliefs are indicated in anxiety and depressive symptoms in youth, but adolescent longitudinal research is required using an age-appropriate metacognitive beliefs measure (i.e., MCQ-A) including at the subscale level.

To address gaps in the research, the current study aims to provide a greater understanding of the relationship between metacognitive beliefs and psychological distress in adolescents, as well as, demonstrate the trajectory of the relationship over time. Psychological distress is operationalised within the current study as nonspecific anxiety and depressive symptoms. Most psychological disorders consist of a combination of somatic distress (somatisation syndromes) and psychological distress. Therefore, the relationship between both phenomena and metacognitive beliefs will be explored [27]. The objective of the current study is to adopt a longitudinal, cohort quantitative research design to explore metacognitive beliefs and psychological distress in adolescents, from the general population, over a 12-month period. Clinical implications include assisting with predicting the aetiology of psychological symptoms in youth, due to the current study being the first known longitudinal analysis exploring the relationship between metacognitive beliefs and psychological distress in adolescents aged 12 to 13. This research could also contribute to the evidence of utilising Metacognitive Therapy within the adolescent population.

The current study postulates that the relationship between psychological distress and metacognitive beliefs, evidenced in the adult population through the S-REF model, will be mirrored within adolescents. Based on the S-REF model, it is anticipated a positive linear relationship will occur between metacognitive beliefs and psychological distress over time. The following hypotheses will be tested:

1. A positive association exists between metacognitive beliefs and the level of psychological and somatic distress in adolescents.
2. Positive and negative metacognitive beliefs are positive predictors of psychological distress in adolescents over a twelve-month period.
3. Metacognitive beliefs reflecting superstition, punishment, and responsibility, cognitive self-consciousness, and low perceived cognitive confidence are positive predictors of psychological distress in adolescents over a twelve-month period.

## Method

### Ethical Approval

This study forms part of a larger project and has received ethics approval from a university human research ethics committee.

### Design

Data were drawn from a larger, longitudinal prospective cohort study. The study recruits community-based participants at age 12, during their first year of high school (Grade 7), across public, private, and independent schools. The study design utilises a rolling recruitment, with each participant assessed at baseline (time-point one) and invited to return for assessment every four months, for approximately five years, until they graduate from high school (Grade 12).

At each time-point, a collection of data is obtained including demographic information, self-report questionnaires, neuropsychiatric interview, cognitive assessment, magnetic resonance imaging (MRI), and electroencephalography (EEG) testing. While the larger longitudinal study was not measuring metacognitive beliefs specifically, the current study was able to implement this variable over four time-points for the first 12 months, enabling the collection of sufficient data to examine associations with psychological distress.

### Participants and Recruitment

Participants were recruited from the greater Sunshine Coast region. Community-based recruitment techniques were utilised, including advertisements via social media platforms (Facebook, University of the Sunshine Coast website), in the local press (print media, television, and radio), and community services (youth groups, headspace, church), as well as engagement with schools. Advertisements encouraged prospective participants, and their guardian(s), to contact the research team if interested in participating in the study.

The inclusion criteria for entry included being in Grade 7 and aged 12 years, 0 months to 12 years, 11 months. Participants were required to be proficient in both spoken and written English. Adolescents with an intellectual disability, major neurological disorder, major medical illness, or those who had sustained a head injury resulting in a loss of consciousness greater than 30 minutes, were excluded due to the possible impact on the ability to complete assessment.

### Procedure

Baseline assessment, including self-report tools, were completed at the time of the first visit, and subsequent assessments were conducted every four months. In all instances, the self-report tools were completed via an electronic questionnaire, under the supervision of a trained research assistant. Participant data obtained outside of the COVID-19 global pandemic was attained in-person at a research lab utilising a touch-screen tablet. Data obtained per the COVID-19 contingency plan occurred online, with questionnaires sent via an email link, and completed during a remote assessment (online video call technology). After assessments, in-person or online, a debriefing procedure was completed.

### Measures

Reliability estimates (i.e., McDonald’s Omega and Cronbach’s alpha) are reported as part of the results using time 1 data in Table 2.

The Kessler Psychological Distress Scale (K10; [28] was utilised in the current study as the primary measure of psychological distress. The K10 is a 10-item tool or assessing symptoms of anxiety and depression during the past four-week period. Frequency-based responses are provided on a five-point Likert scale. An example item includes: “In the past four weeks, about how often did you feel worthless?” Participant total scores range from 10 to 50, with higher scores representing increased levels of psychological distress. Research has reported strong associations between high scores on the K10 and diagnoses of anxiety and affective disorders [29]. The K10 has been validated for use in adolescents, aged 12 to 19 years [30].

The Somatic and Psychological Health Report (SPHERE-12; [27] was included to allow for the possibility of independent symptom trajectories across psychological distress and somatic distress in adolescents [31]. The SPHERE-12 can be analysed at a subscale level. Subscale one (PSYCH), represents psychological distress, including common symptoms of anxiety and depression. Subscale two (SOMA), measures somatic distress, for example, fatigue. Item scores range from 0 (*never or some of the time*) to 2 (*most of the time*), with subscale scores ranging from 0 to 12.

The Meta-Cognitions Questionnaire for Adolescents (MCQ-A; [8], is a 30-item measure of metacognitive beliefs,validated for use in adolescents aged 13 to 17. Factor one, PBW, assesses for positive metacognitive beliefs, an example item includes “worrying helps me avoid problems in the future.” Factor two, NBW, measures negative metacognitive beliefs; for example, “my worrying could make me go mad.” Factor three, CC, measures an individual’s perceived lack of confidence in their attention and memory; for example, “I have little confidence in my memory for words and names.” Factor four, SPR, assesses for negative consequences of not controlling thoughts; for example, “if I did not control a worrying thought, and then it happened, it would be my fault.” Lastly, factor five, CSC, assesses the tendency to focus attention on thought processes; for example, “I monitor my thoughts” [8]. Participants specify how much they agree with each statement on a four-point scale, ranging from “do not agree” to “agree very much”. Subscale scores range from 6 to 24 and the total MCQ-A score is a cumulation of the five subscales.

### Statistical Analysis

All analyses were conducted using SPSS Version 26. Associations between metacognitive beliefs and psychological distress were tested using Spearman’s rho correlational analyses for the K10 total score, SPHERE-12 PSYCH subscale, and MCQ-A total score, as well as MCQ-A subscales, at time-points 1–4. Spearman’s rho correlational analyses of the SPHERE-12 SOMA subscale, MCQ-A total score, and MCQ-A subscales were analysed to gauge relationships between metacognitive beliefs and somatic distress at time-points 1–4. Results were interpreted as statistically significant if p < 0.05.

Generalised Estimating Equations analyses were produced to test hypotheses two and three. Generalised estimating equations is a method often used in longitudinal data and has been used in sample sizes of 83–89 to explore longitudinal associations of anxiety and depression [32]. Using a significance criterion of α = 0.05 and power = .80, a sample of 92 was established to detect a medium effect size (Faul et al., 2007). Unfortunately, the current study’s participant numbers reduced from time-point 1–4, reflective of the ongoing nature of a longitudinal study and the rolling recruitment design. Thus, generalised estimating equations was selected as the planned analysis as it does not require each participant to have completed data at every time-point [33] and may still be applied to sample sizes ≥50 [34]. Based on normality testing, the Generalised Estimating Equations Gamma log link under exchangeable correlation structure was specifically utilised.

Generalised estimating equations is a semi-parametric research method, and therefore all data distributions are supported [35]. Across the five generalised estimating equations analyses, the applicable MCQ-A subscale was entered as the predictor variable, and the K10 total score was entered as the dependent variable. Regression coefficients with corresponding 95% confidence intervals were calculated. Univariate generalised estimating equations analyses demonstrate the association between the metacognitive predictor variable and psychological distress, over the four time-points, simultaneously. Significant results are reported as the exponential of the estimated regression coefficient for the outcome variable. A model was built for each of the five metacognitive factors. Quasi-likelihood under the independence model criterion (QIC) was used for each model to identify the best model fit.

## Results

Demographic statistics across the four time-points analysed are detailed in Table 1. Participant numbers range from 70 (time-point 1) to 53 (time-point 4). Descriptive statistics across the analysed outcome measures are reflected in Table 2 to provide an indication of the level of psychological distress and metacognitive beliefs experienced by the current sample. The mean total scores for psychological distress (K10) across the four time-points indicate that as a group the current sample is within the well range (*M* = 15.13, *SD* = 5.44).

**Table 1:** Demographic statistics

	TP1	TP2	TP3	TP4
Participants	*n* = 70	*n* = 61	*n* = 55	*n* = 53
Gender (female)	31 (44.3%)	29 (47.5%)	26 (47.3%)	25 (47.2%)
Gender (male)	39 (55.7%)	32 (52.5%)	29 (52.7%)	28 (52.8%)
Age in years, Mean (SD)	12.65 (0.31)	12.98 (0.33)	13.33 (0.34)	13.70 (0.33)
Grade, Mean (SD)	7.00 (*SD* 0.00)	7.44 (0.50)	7.93 (0.26)	8.00 (0.00)

*TP* Time-point

**Table 2:** Descriptive statistics for measures

Variable	Reliability Estimates		TP1	TP2	TP3	TP4
	ω	α	*M (SD)*	*M (SD)*	*M (SD)*	*M (SD)*
MCQ-A	0.79	0.84	57.31 (10.97)	51.36 (10.51)	51.78 (11.14)	52.43 (11.79)
PBW	0.90	0.89	8.94 (3.39)	8.30 (3.08)	8.89 (3.39)	9.09 (3.47)
NBW	0.83	0.83	10.97 (3.52)	9.85 (3.13)	9.44 (2.79)	9.66 (3.37)
CC	0.77	0.76	10.57 (3.34)	9.23 (3.00)	9.82 (3.18)	9.58 (3.40)
SPR	0.52	0.53	11.99 (3.16)	10.44 (2.99)	10.00 (2.78)	10.25 (2.79)
CSC	0.74	0.74	14.84 (3.22)	13.54 (3.85)	13.64 (4.39)	13.85 (4.51)
K10 Total	0.83	0.86	15.74 (5.30)	14.69 (5.36)	14.64 (4.23)	15.34 (6.75)
SPHERE (Psych)	0.80	0.80	1.33 (1.99)	0.75 (1.42)	0.69 (1.14)	1.04 (1.84)
SPHERE (Soma)	0.77	0.77	2.64 (2.49)	1.84 (2.30)	1.60 (1.89)	2.06 (2.54)

*ω* Omega (total) for time 1 data, *α* Coefficient alpha for time 1 data, *TP* Timepoint, *MCQ-A* Metacognitive Questionnaire for Adolescents Total Score, *PBW* Positive Beliefs about Worry, *NBW* Negative Beliefs about Worry, *CC* Cognitive Confidence, *SPR* Superstition, Punishment and Responsibility, *CSC* Cognitive Self-Consciousness, *K10* Kessler Psychological Distress Scale, *PSY* SPHERE-12 (PSYCH) subscale, *SOMA* Somatic and Psychological Health Report (SOMA) subscale for somatic symptoms

Assumptions of normality, linearity, and homoscedasticity were assessed. The Shapiro-Wilk test of normality was violated (*Sig* < .05). Therefore, Spearman’s rho was selected to analyse correlational relationships. All subscales of the MCQ-A demonstrated significant positive correlations with each other and the total score, ranging from rs = .19 to rs = .76, all *ps* < .05.

Hypothesis one was supported. A statistically significant positive correlation was found between total level of metacognitive beliefs and psychological distress measured by the K10, at time-point 1 [rs (.48), *p* < .001, two-tailed, *N* = 70], time-point 2 [rs (.40), *p* = .01, two-tailed *N* = 61], time-point 3 [rs (.58), *p* = .01, two-tailed *N* = 55], and time-point 4 [rs (.48), *p*< .001, two-tailed *N* = 53]. Refer to Table 3 for the Spearman rho correlations for MCQ-A total score, associated subscales, and the K10.

**Table 3:** Analysis of hypothesis one using spearman’s rho (Psychological Distress)

	TP1		TP2		TP3		TP4	
	K10	PSY	K10	PSY	K10	PSY	K10	PSY
MCQ- A	0.48***	0.55***	0.40**	0.51***	0.58**	0.28*	0.48***	0.55***
PBW	0.36**	0.35**	0.21	0.17	0.28*	0.44**	0.42**	0.41**
NBW	0.48***	0.46***	0.37**	0.46***	0.50**	0.43**	0.41**	0.50***
CC	0.44***	0.22	0.49***	0.46***	0.46**	0.31*	0.40**	0.48***
SPR	0.23	0.37**	0.39**	0.41**	0.47**	0.27*	0.39**	0.48***
CSC	0.04	0.29*	0.07	0.19	0.36**	0.22	0.17	0.26

*TP* Time-point, *K10* Kessler Psychological Distress Scale, *PSY* SPHERE-12 (PSYCH) subscale, *MCQ-A* Metacognitive Questionnaire for Adolescents Total Score, *PBW* Positive Beliefs about Worry, *NBW* Negative Beliefs about Worry, *CC* Cognitive Confidence, *SPR* Superstition, Punishment and Responsibility, *CSC* Cognitive Self-Consciousness

^***^Significant difference at p < 0.001, **Significant difference at p < 0.01, *Significant difference at p < 0.05

Hypothesis one was also supported when comparing the relationship between total level of metacognitive beliefs and psychological distress measured by the SPHERE-12 (PSYCH) subscale at time-point 1 [rs (.55), *p* < .001, two-tailed, *N* = 70], time-point 2 [rs (.51), *p*< .001, two-tailed *N* = 61], time-point 3 [rs (.28), *p* = .01, two-tailed *N* = 55], and time-point 4 [rs (.55), *p* < .001, two-tailed *N* = 53]. The findings across the two measures, when compared to metacognitive beliefs, demonstrate the consistency of the relationship. At a subscale level, NBW had the strongest positive relationship with psychological distress in the sample.

In further support of hypothesis one, Spearman’s rho revealed a statistically significant positive correlation between total level of metacognitive beliefs and somatic distress at time-point 1 [rs (.48), *p* < .001, two-tailed, *N* = 70], and time-point 4 [rs (.33), *p* = .05, two-tailed, *N* = 53], only. There was no significant findings found for the overall measure of metacognitive beliefs and somatic distress at time-point 2 or 3, as detailed in Table 4. Limited and inconsistent findings were found at the subscale level of metacognitive beliefs.

**Table 4:** Analysis of hypothesis one using spearman’s rho (Somatic Distress)

	TP1	TP2	TP3	TP4
	SOMA	SOMA	SOMA	SOMA
MCQ-A	0.48***	0.14	0.24	0.33*
PBW	0.25*	0.12	0.21	0.28*
NBW	0.35**	0.10	0.24	0.30*
CC	0.38**	0.42**	0.21	0.30*
SPR	0.41***	0.22	0.13	0.31*
CSC	0.16	− 0.14	0.07	0.12

*TP* Time-point, *SOMA* Somatic and Psychological Health Report (SOMA) subscale for somatic symptoms, *MCQ-A* Metacognitive Questionnaire for Adolescents Total Score, *PBW* Positive Beliefs about Worry, *NBW* Negative Beliefs about Worry, *CC* Cognitive Confidence, *SPR* Superstition, Punishment and Responsibility, *CSC* Cognitive Self-Consciousness

^***^Significant difference at p < 0.001, ** Significant difference at p < 0.01, * Significant difference at p < 0.05

Generalised estimating equations was utilised to analyse hypotheses two to three and produced models of correlated data across the 12-month period. The assumption of normality was violated. Therefore, generalised estimating equations with gamma log link under exchangeable correlation was used. Table 5 details univariate results of metacognitive predictors of psychological distress (K10), over a 12-month period.

**Table 5:** Generalised estimating equations univariate analyses over a 12-month period

Metacognitive predictor	DV	B	95%	CI	Wald chi square	df	Sig
			Lower	Upper			
PBW	K10	0.02	0.01	0.04	10.77	1	*p* = 0.001*
NBW	K10	0.05	0.04	0.06	64.81	1	*p* < 0.001*
CC	K10	0.04	0.03	0.06	45.83	1	*p* < 0.001*
SPR	K10	0.04	0.03	0.05	40.94	1	*p* < 0.001*
CSC	K10	0.01	0.00	0.02	4.54	1	*p* = 0.033*

*DV* Dependent Variable, *K10* Kessler Psychological Distress Scale, *CI* Confidence Interval, *PBW* Positive Beliefs about Worry, *NBW* Negative Beliefs about Worry, *CC* Cognitive Confidence, *SPR* Superstition, Punishment and Responsibility, *CSC* Cognitive Self-Consciousness

^*^Significant at p < .05

Hypothesis two was supported, and there is a significant prediction from the level of PBW to the level of psychological distress. Over the twelve-month period, a 1 unit increase in PBW resulted in a .02 increase in psychological distress.

Similarly, NBW found to be a significant predictor of psychological distress in adolescence over a twelve-month period. Specifically, after allowing for multiple time-points, results showed that for each unit increase in NBW, psychological distress increased by an estimated .05. Furthermore, based on the model-fit indices reported in Table 6, the correlational structure between NBW and psychological distress represents the best model fit of the five models produced.

**Table 6:** Generalised estimating equations model fit

Metacognitive predictor	DV	Model fit
PBW	K10	QIC = 31.31, QICC = 26.75
NBW	K10	QIC = 24.49, QICC = 20.60
CC	K10	QIC = 26.51, QICC = 22.57
SPR	K10	QIC = 28.11, QICC = 24.67
CSC	K10	QIC = 30.98, QICC = 27.78

*DV* Dependent Variable, *K10* Kessler Psychological Distress Scale, *PBW* Positive Beliefs about Worry, *NBW* Negative Beliefs about Worry, *CC* Cognitive Confidence, *SPR* Superstition, Punishment and Responsibility, *CSC* Cognitive Self- Consciousness, *QIC* Quasi Likelihood under Independence Model Criterion, *QICC* Correct Quasi Likelihood under Independence Model Criterion

Hypothesis three was supported with SPR and CC found to be significant predictors of psychological distress each resulting in an increase of .04. Likewise, CSC was found to be a significant predictor of psychological distress resulting in an increase of .01. The CSC subscale represented the weakest relationship with psychological distress.

## Discussion

The current study aimed to provide a greater understanding of the relationship between metacognitive beliefs and psychological distress in adolescents, as well as provide an indication of the course over time. Results suggest that a community sample of early adolescents endorse a range of metacognitive beliefs associated with psychological distress, thus, contributing to the emerging support for the utility of the S-REF model in adolescence.

Correlational analyses were conducted across four time-points to assess hypotheses one. Hypothesis one was supported, and a significant positive relationship between the level of metacognitive beliefs and psychological distress occurred across all four time-points. This relationship mirrors that seen within the adult population in regard to the S-REF, which has found that higher metacognitive beliefs correlates with higher endorsement of perceived stress and anxiety symptomology [9, 36, 37]. At a subscale level, NBW had a significant relationship with psychological distress across all four-time points. This finding is consistent with reported associations between NBW and psychological symptoms in research exploring non-clinical populations in adolescents [22, 23]. Moreover, substantial research has established that negative metacognitive beliefs are the most pervasive and potent metacognitive beliefs in psychological disorder [7]. Longitudinally, our finding contributes to the emerging understanding that negative metacognitive beliefs are pivotal in adolescent mental health and support the S-REF theoretical model for this age group.

Regarding the hypothesized positive association between metacognitive beliefs and the level of somatic distress in adolescents, mixed results were found; indicating the relationship in adolescents is unclear. Potentially, these results may support previous research suggesting somatic distress and psychological distress can have different courses within the adolescent population [31]. Alternatively, the results may indicate metacognitive beliefs increase psychological symptoms of anxiety and depression, such as a sense of hopelessness, more than somatisation (e.g., fatigue, pain) in adolescents. Further, the findings may be due to methodological limitations of the current study (i.e., small sample size, community-based sample).

Nonetheless, all five metacognitive constructs (PBW, NBW, CC, SPR, and CSC) were found to be significant predictors of psychological distress, as measured by the K10, in adolescents aged 12 to 13 over 12 months (four time-points). This provides theoretical support for the S-REF model and is coherent with Cartwright-Hatton et al. [8] finding in the development of the MCQ-A that the “full range of meta-cognitive beliefs that have been identified in adult populations were also endorsed by adolescents” (p. 420).

In regard to PBW, these metacognitive beliefs significantly predicted psychological distress in adolescents over a 12-month period. Cross-sectional research has found that PBW are higher in adolescents with anxiety symptoms, compared to non-clinical controls [17]. Our findings build upon this research by estimating a positive linear trajectory of PBW and psychological distress in adolescents. Consistent with the S-REF, this may be reflective of the influencing role of PBW in driving engagement in worry and, in turn, maladaptive coping strategies leading to the onset of psychological distress in youth. Similarly, other longitudinal research in university students found a significant indirect effect of positive beliefs about rumination increased the use of rumination, resulting in elevated depressive symptoms over-time, in a non-clinical adult sample [38].

Longitudinally, negative metacognitive beliefs were a predictor of psychological distress over a twelve-month period. Concordant with research on negative metacognitive beliefs and the S-REF, NBW were the strongest predictor of psychological distress identified in the current study and the longitudinal model had the best fit. This highlights that NBW may contribute most to the development and onset of psychopathology in early adolescence. This finding supports the S-REF model and the role of NBW in activating the Cognitive Attentional Syndrome including repetitive pervasive thinking [5, 39]. These results mirror adult longitudinal research. In a prospective study utilising a two-time measurement design, within a non-clinical adult population, NBW predicted depression and anxiety symptoms, independent of life events [40]. Therefore, the pattern of results in the current adolescent study appears consistent with the adult population. Indicating the relevance of the S-REF model and metacognitive therapy for adolescent mental health.

Lower levels of CC (i.e., higher scores on the CC subscale equate to lower cognitive confidence; “I have a poor memory”, “I do not trust my memory”) were a significant predictor of psychological distress over time in adolescents aged 12 to 13. Similarly, in the current research trial with a different configuration of participants, low CC was a mediator between cybervictimization and quality of life [41]. Interestingly, in adults over time, low CC has been noted to interact with experiencing daily stressors, including interpersonal conflicts and social rejection to predict anxiety [40]. Our results are consistent with Yilmaz’s findings given the current study assessed adolescents across their commencement of high school, which likely increased demand on the allocation of cognitive resources (e.g., attention) and exposure to social challenges, and everyday stressors. This finding contributes to the scarce research on CC in adolescents. To date, Ellis and Hudson [17] found no difference in between clinically anxious and non-anxious adolescents with CC,whereas, Cartwright-Hatton et al. [8] found clinical youth had low CC.

Over a twelve-month period, SPR metacognitive beliefs were a predictor of psychological distress. This finding regarding SPR metacognitive beliefs extends upon cross-sectional research in the adolescent population which has found a positive relationship between SPR and psychological symptoms, specifically obsessive-compulsive symptoms [18, 23, 24]. Similarly, within the adult population, metacognitive beliefs related to the need to control thoughts were higher amongst individuals meeting diagnostic criteria for Obsessive-Compulsive Disorder and Panic Disorder, compared to non-clinical participants [10]. Therefore, as seen in adult research, holding beliefs regarding the need to control thoughts to prevent adverse outcomes may predispose adolescents to psychological distress [10].

Metacognitive beliefs relating to CSC in adolescents over a twelve-month period were a significant predictor of psychological distress in the current study; however, it represented the weakest association over-time. This finding aligns with the growing evidence in adolescent populations suggesting monitoring one’s thoughts is a normative process amongst youth and that CSC may be less implicated in terms of psychological conditions but this needs further evaluation [8, 15–17].

The results of this study have several clinical implications. Firstly, the study builds upon the existing research regarding the role of metacognitive beliefs and psychological distress in adolescents. Notably, the study has also provided an insight into the possible trajectory and direction of the relationship. No previous known study has analysed the longitudinal relationship between metacognitive beliefs and psychological distress in an exclusively adolescent population of 12 to 13-year-old participants and used an age-appropriate measure of metacognitive beliefs (i.e., MCQ-A). Therefore, the current study provides a unique insight into the metacognitive processes at this critical age.

The current results found support for the role of all five metacognitive beliefs in psychological distress in a community sample of adolescents aged 12-to-13-years. This supports Wells’ metacognitive theory of psychological disorder and the S-REF model. Our findings endorse the role of metacognitive beliefs and their relationship to psychological distress, including much needed longitudinal evidence. However, it is essential that future research in adolescent mental health expands our findings by including specific measures related to the cognitive-attentional syndrome (e.g., worry or rumination frequency). This will elaborate further on the role of metacognitive beliefs in driving and maintaining the cognitive-attentional syndrome (i.e., worry) in adolescents’ emotional distress.

The study has important methodological limitations that need consideration. The study consists of a community-based sample of adolescents, which provides a barrier to the generalisability of the findings to clinical adolescent populations. The nonprobability sampling method also results in a self-selection bias. Participants may share distinctive characteristics, further limiting the study’s generalisability. The data assessed within the current study did not meet normality assumptions for parametric data analysis which imposed an additional limitation. A semi-parametric analysis, generalised estimating equations, was utilised to analyse longitudinal data, which limits the current findings to providing a preliminary indication of the relationship between metacognitive beliefs and psychological distress in adolescence over time. Further, the small sample size and missing data across time-points limit the findings of the current study and caution is required interpreting statistically significant results. Taken together, while generalized estimating equations is robust against model misspecification (Hubbard et al., 2010), future research may seek to replicate the current findings with larger samples and suitable parametric analyses (e.g., path analsysi, cross-lagged panel model). Finally, the SPHERE-12 was only used for hypothesis one and not used in the generalised estimating equations analyses due to the sample size, future longitudinal research should consider utilising a somatic measure such as the SPHERE-12.

Another limitation relates to the outcome measure utilised for metacognitive beliefs. The MCQ-A is validated in adolescents aged 13–17, and the current study reported on adolescents aged 12 to 13. An alternative measure, the Metacognitive Questionnaire for Children (MCQ-C), incorporates a more comprehensive age range, 7–17 years. However, the Metacognitive Questionnaire for Children does not assess for CC, a metacognitive process which already presents a gap in the literature regarding its role in youth mental health. Lastly, research by Bacow et al. [16] found that female adolescents within their clinical population, presenting with an anxiety disorder, endorsed higher overall levels of metacognitive beliefs. Therefore, a limitation of the current study is that the role of gender effects was not assessed.

Future longitudinal studies with larger sample sizes are required to build upon the current preliminary findings. Adolescence is a period of marked cognitive and psychological development [16]. Therefore, the course of the trajectory between metacognitive beliefs and psychological distress should be extended to explore adolescents aged 12 to 17. To further demonstrate the role of the S-REF in adolescence, future research is required in clinical samples to determine whether the unique metacognitive beliefs and CAS processes (e.g., worry) demonstrated within the adult population for certain psychological diagnoses are reflected within youth.

In conclusion, this study contributes to the existing body of research by being the first known longitudinal study to explore the relationship between metacognitive beliefs and psychological distress in an exclusively adolescent population aged 12 to 13. The results support the applicability of the S-REF model in young people and found metacognitive beliefs predict psychological distress in early adolescence over time. NBW emerged as a significant predictor of distress in adolescents and being aware of this vulnerability may allow for the early detection of subclinical manifestations in youth to facilitate early intervention. The current findings contribute to the rationale for psychological treatment to target metacognitive beliefs in adolescents presenting with psychological distress [42].

## Summary

The metacognitive model of psychological disorders may assist in understanding the onset and maintenance of psychological disorders across the lifespan. Accordingly, the current study extended upon previous research by investing the longitudinal relationships of metacognitive beliefs and psychological distress in a community-based sample of adolescents, aged 12–13. Self-report measures of metacognitive beliefs, psychological distress, and somatic distress were obtained across four time points over 12 months. Correlational analyses demonstrated a significant relationship between overall metacognition, as well as negative metacognitive beliefs, and psychological distress across all time points. Moreover, Generalised Estimating Equations supported significant associations between different various metacognitive predictors and psychological distress across all time points. Negative metacognitive beliefs appeared to have the strongest relationship with psychological distress. Collectively, the findings provide preliminary evidence for the importance of metacognitive beliefs in the aetiology of psychological distress in adolescents aged 12–13, congruent with metacognitive theory of psychological disorder and the S-REF model. The findings also encourage further research regarding psychological interventions targeting metacognitive beliefs in adolescents presenting with symptoms of psychological distress.

## Data Availability

Data are not available for distribution.
